# 

**Published:** 1887-12-31

**Authors:** 


### CONTENTS.

**Home Rule for Nurses** - 227

**Words of Consolation.**—The End and the Beginning - 228

**Retrospect** - 228

**The Noblest Monument of the Queen's Reign.** By Sir ALBERT ROLLIT, M.P. - 229

**Amusements for Convalescents.**—Palmistry Notes. By A LADY - 230

**The Doctor's Pulpit.**—Men and Marriage - 231

**Comparative Expenditure of Various Hospitals for the Year 1886** - 232

**Clinical nd Therapeutic Notes.** By C. R. ILLINGWORTH, M.D., M.R.C.S.—Pyrexia and Anti-pyretics - 233

**Notes and News** - 234

**Annotations.**—Progress in Co-operation.—St. John's Hospital for Diseases of the Skin.—Children's Dress, etc. - 237

**Common Accidents of Every-day Life.** By R. A. P. FORRESTER, B.A., Dub.—Burns and Scalds - 238

**A Ghastly Experience** - 239

**Incidents of Animal Life** - 240

**The Students' Column.**—University of London - 241

**The Editor's Letter-Box.**—Recollections of Hospital Life - 241

**Christmas Entertainments at Hospitals** - 241

**Amusements with Profit for all Ages.**—Competitions, Puzzles, Children's Corner, etc. - 242

NOTICE.—"THE HOSPITAL" ALMANACK, 1888, WILL BE GIVEN GRATIS WITH NEXT WEEK'S ISSUE.

